# Acoustotactic response of mosquitoes in untethered flight to incidental sound

**DOI:** 10.1038/s41598-021-81456-5

**Published:** 2021-01-21

**Authors:** Zhongwang Dou, Aditi Madan, Jenny S. Carlson, Joseph Chung, Tyler Spoleti, George Dimopoulos, Anthony Cammarato, Rajat Mittal

**Affiliations:** 1 Department of Mechanical Engineering, Whiting School of Engineering, Johns Hopkins University, Baltimore, MD USA; 2 Division of Cardiology, Department of Medicine, School of Medicine, Johns Hopkins University, Baltimore, MD USA; 3 Department of Molecular Microbiology and Immunology, Bloomberg School of Public Health, Johns Hopkins University, Baltimore, MD USA

**Keywords:** Mechanical engineering, Malaria, Animal behaviour

## Abstract

Mosquitoes are vectors for some of the most devastating diseases on the planet. Given the centrality of acoustic sensing in the precopulatory behavior of these vectors, the use of an exogenous acoustic stimulus offers the potential of interfering with the courtship behavior of these insects. Previous research on the acoustotactic response of mosquitoes has been conducted on tethered preparations using low-intensity sound stimuli. To quantify differences in acoustotactic responses between mosquitos of distinct sex and species, we examined the effects of incidental sound stimuli on the flight behavior of free-flying male vs. female *Aedes aegypti* and *Anopheles gambiae* mosquitoes. The key variables were sound frequency (100–1000 Hz) and intensity (67–103 dB, measured at 12.5 cm from the source), and the acoustotactic response was measured in terms of the relative increase in flight speed in response to the stimulus. The data show, for the first time, significant sex- and species-specific differences in acoustotactic responses. *A. aegypti* exhibited a greater response to sound stimulus compared to *An. gambiae*, and the response also extended over a larger range of frequencies. Furthermore, the males of both species displayed a greater acoustotactic response than females, with *An. gambiae* females exhibiting minimal response to sound.

## Introduction

Mosquitoes are vectors for a variety of potentially fatal diseases, including malaria, Zika fever, dengue, and chikungunya^1^. During courtship, mosquitoes are known to exploit wing-tones (i.e., sounds from flapping wings) to recognize conspecifics, display fitness, and transmit mating interest^2–7^. This intricate aerial communication is facilitated by the exceptional sensitivity of the Johnston’s Organ (JO)^8^, which in male mosquitoes, contains 15,000 primary neurons^9–11^ compared to < 500 in the similar-sized *Drosophila*^12^. Studies have shown that the JO of mosquitoes is “tuned” to frequencies associated with these wing-tones, further emphasizing the criticality of flight-tone based signaling in the courtship behavior of mosquitoes during their lifecycle.

Given the exceptional sensitivity of mosquitoes to the flight tones of conspecifics, it has been postulated that exposure of mosquitoes to exogenous sounds with appropriate frequencies could modify their flight behavior, precopulatory communication, and mating success^3,4^, and in doing so, potentially reduce the reproductive rates of these disease vectors. While much of this line of research is unfortunately riddled with pseudoscience^13–17^, recent successes in using sound and flight-tone based approaches to survey/trap mosquitoes^18–23^ vindicate many thorough scientific investigations, some going back 60 + years, which have explored the effects of sound on mosquito behavior^6,18,24,25^.

A well-characterized behavior in this arena is the phenomenon of “acoustic startle” in mosquitoes studied by Gibson and Russell^5^, where they observed a transient and rapid increase in *Toxorhynchites brevipalpis* wing-beat frequency (WBF) in response to exogenous acoustic tones in the frequency range 350–490 Hz and intensity > 90 dB Sound Pressure Level (SPL) (measured 3 cm from the loudspeaker). They also observed a similar response for the frequency ranges between 200 and 345 Hz and 500–800 Hz, but at a lower intensity of 40–65 dB SPL.

The study of Gibson and Russell^5^ as well as most other studies^6,7^ used tethered mosquito preparations where individual animals were adhered by their dorsal thorax to a ~ 100 μm long stainless steel wire, which was then mounted to a micro-positioner to ease behavioral assessment. However, it is well known that tethering can change wing movements and responses of these insects^26^. Furthermore, the free flight response to an acoustic stimulus obviously cannot be quantified when insects are tether-restricted. Finally, to the best of knowledge, response differences between mosquito species and sexes have never been evaluated in tethered or free-flying animals.

In the current study, we have, for the first time, elucidated the effects of incidental acoustic waves on groups of free-flying mosquitoes. Importantly, our current focus is not on understanding precopulatory acoustic interactions between conspecifics, such as in the aforementioned investigations, but to quantitatively compare the effects of incidental sound on the flight behavior of free-flying mosquitoes of different species and sexes, over a wide range of acoustic frequencies and intensities. We utilized high-speed videogrammetry to record the motion of free-flying mosquito groups, before and after the application of exogenous acoustic stimuli. The study was conducted using male or female *Aedes aegypti* (*A. aegypti*) or *Anopheles gambiae (An. gambiae)*. The acoustotactic response was assessed via changes in flight speed and quantified over a range of acoustic frequencies and intensities. Our results show significant sex- and species-specific differences in acoustotactic responses of a group of free-flying mosquitoes.

## Results

We employed a high-speed camera (IDT Y4-S1, 1024 × 1024 pixel, 500 frame-per-second (FPS) at 125 μm/pixel during recording) to extract the in-plane velocity of free-flying mosquitoes. For each 2-s recording, the exogenous sound was turned on immediately after the first second of the recording. The two-dimensional velocity of mosquito flight versus time was obtained after post-processing of the video, as described in the “Methods” section. The average flight speed before the onset of the acoustic wave was calculated as 0.14–0.18 m/s for all test cases recorded here. When the sound was turned on, we observed measurable changes in flight speed, which depended on the species and sex of the mosquito as well as the acoustic frequency and intensity.

### Effect of sound frequency

To investigate the effect of frequency on the acoustotactic response, we recorded high-resolution videos of all four groups of free-flying mosquitoes and tested reactions to sound frequencies ranging from 100 to 1000 Hz (Table 2 in the “Methods” section) with 100 Hz increments. The sound intensity for this set of experiments was set at 103 dB, the highest intensity utilized for all experiments (see below), measured at the center of the mosquito cage, which was located at a distance of 12.5 cm from the speaker. We picked the highest acoustic intensity to study the effect of sound frequency to ensure a high likelihood of observing a measurable response. Subsequent tests explore the effect of sound intensity on the response.

**Figure 1:**
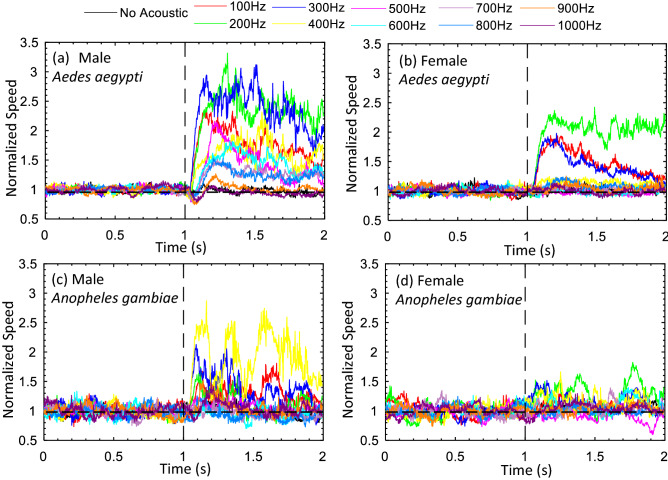
Acoustic wave effect on the flight speed of flying mosquitoes at different frequencies at a sound intensity of 103 dB. The flight speed results were normalized by the average speed obtained during the 1st second in each test. (**a**), (**b**), (**c**), and (**d**) were normalized flight speeds of male and female *Aedes*, and male and female *Anopheles*, respectively.

**Figure 2:**
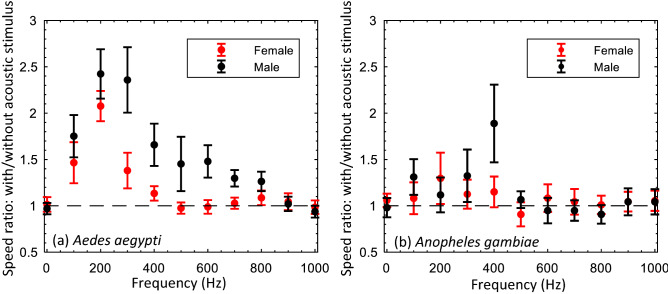
The ratio of mosquito flight with/without acoustic wave at different acoustic frequencies at a fixed sound intensity of 103 dB. (**a**) *Aedes* and (**b**) *Anopheles*.

**Figure 3:**
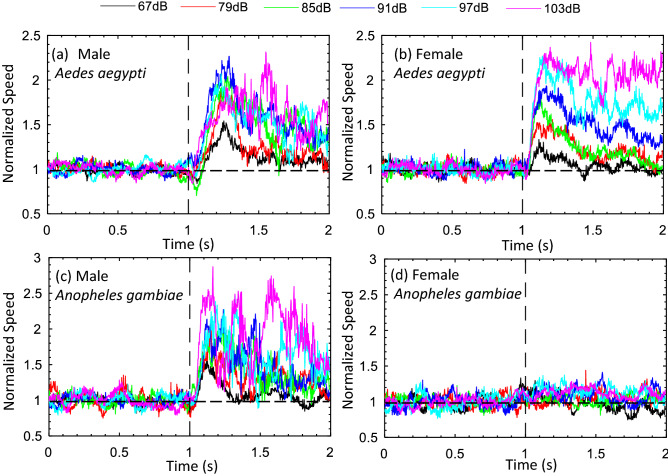
Effect of acoustic stimulus on the flight speed of mosquitoes at different intensities. The sound frequency was kept fixed at 400 Hz for all male mosquitoes and 200 Hz for the female mosquitoes. The flight speed results were normalized based on the average speed obtained from the 1st second in each test. (**a**), (**b**), (**c**), and (**d**) are normalized flight speeds of male *Aedes*, female *Aedes*, male *Anopheles*, and female *Anopheles*, respectively.

**Figure 4:**
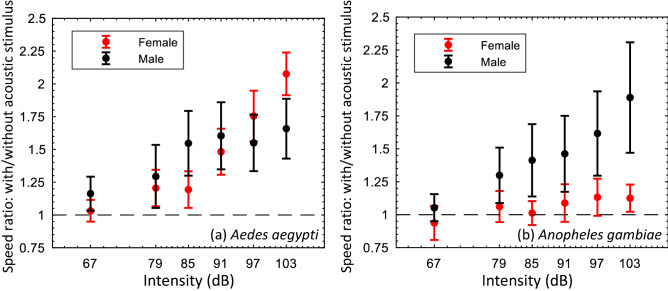
The ratio of mosquito flight speed with/without an acoustic stimulus, at different acoustic intensities, at a fixed frequency of 400 Hz for males and 200 Hz for females. (**a**) and (**b**) are for *Aedes* and *Anopheles*, respectively.

For each test condition, six independent experiments were carried out. Figures 1 and 3 show flight speeds, ensemble averaged over the six independent experiments, as a function of time. In these plots, the speed after the stimulus was normalized by the average flight speed before the acoustic stimulus was turned on. Figures 2 and 4 show the corresponding ensemble averaged ratio of flight speed with and without acoustic stimulus, including the variance in the six experiments.

*Aedes* males exhibited an increase in flight speed over a broad range of sound frequencies between 100 and 800 Hz (Figs. 1a, 2a). The greatest response was observed at 200 and 300 Hz. For these two frequencies, the flight speed increased by just over 135%. Within the statistical variance of our measurements, the acoustotactic response at these two frequencies was virtually indistinguishable. In most cases for which a measurable increase in speed was observed, the response of *Aedes* males attained its maximum approximately 200 ms after stimulus onset and was observed to decay subsequently. The rate of decay was slower for frequencies that elicited a strong response. For instance, at a stimulus frequency of 300 Hz, the normalized speed dropped from a peak of about 2.75 to about 2.0 (i.e., a 27% reduction) over the duration of a second whereas for a sound frequency of 100 Hz, the normalized flight speed reduced from a peak of about 2.3–1.5 (i.e., a 35% reduction).

*Aedes* females responded to a narrower range of acoustic frequencies that extended between 100 and 300 Hz. The most effective frequency for eliciting an acoustotactic response from *Aedes* females was 200 Hz, where the flight speed increased by 110% (Figs. 1b, 2a). At this frequency, the flight speed after the stimulus was initiated, dropped from a peak of 2.25 to about 2.1 (i.e., about 7%) in 1 s. The time to peak response was also about 200 ms for most cases.

For *Anopheles* males, altered flight speed was observed in a frequency range of 100 to 400 Hz, with the most prominent response at 400 Hz, which elicited a 90% increase in average flight speed (Figs. 1c, 2b). The decay rate of the response was difficult to estimate given the high variability in the ensemble-averaged data, but peak values of normalized flight speed increases at 400 Hz were as high as 2.5, and dropped to about 1.5 in a second. The time to peak response was 100 ms post-stimulus initiation.

Finally, for *Anopheles* females, the acoustotactic response (Figs. 1d, 2b) was also limited to a range of frequencies from 100 to 400 Hz. The maximum response was at a frequency of 200 Hz, where the flight speeds increased by about 30% (Fig. 2b). For *Anopheles* females, the response at 200 Hz was slightly elevated over the 2 s. Finally, the time to peak response after the initiation of the stimulus was difficult to accurately ascertain, but was 100 ms for the cases for which a measurable response was noted.

### Effect of sound intensity

To study the effect of the intensity of sound on the acoustotactic response, we subjected all four groups of free-flying mosquitoes to a range of sound intensities (Table 3 in the “Methods” section) at a fixed frequency. To make the comparison meaningful, we thought it was necessary to select the same fixed frequency for both species in this study. Since female *An. Gambiae* only responded at 200 Hz while *A. Aegypti* responded between 100 and 300 Hz, we picked 200 Hz as the common frequency for the females. Similar considerations led to the choice of 400 Hz as the test frequency for the males. The normalized flight speeds were plotted against time in Fig. 3. We also plotted the ratio of normalized flight speed with and without acoustic stimulus against the acoustic intensity averaged over six independent experiments in Fig. 4.

For *Aedes* males, the effect of sound intensity on flight speed increased with increasing intensity from 65 dB (15% increase in flight speed) to 85 dB (60% increase in flight speed) (Figs. 3a, 4a). Increased sound intensity, beyond 85 dB and up to 103 dB, did not affect the response significantly, and we observed about a 60% increase in flight speeds for all intensities. For the lowest intensity of 67 dB, the response peaked at a normalized flight speed value of 1.5 within 275 ms of stimulus onset, but decayed to a value of 1.1 around 225 ms. At a sound intensity of 91 dB, the peak value of normalized flight speed was 2.25 approximately 225 ms after the initiation of the stimulus and decayed to 1.3 over a duration of 775 ms.

Interestingly, *Aedes* females exhibited a response that was the opposite of that of the *Aedes* males. An initial increase in intensity from 65 to 85 dB resulted in a small (20%) speed increase but beyond 85 dB, the response grew rapidly with increasing intensity, and reached 110% increase at 103 dB (Figs. 3b, 4a). The eventual decay in the response was more monotonic compared to the males. For the lowest intensity stimulus of 67 dB, the response decayed from its peak of 1.3 to nearly 1.0 in a duration of about 400 ms. For a sound intensity of 91 dB, the response continuously decayed from a peak value of 1.9 at 175 ms after the stimulus, to a value of 1.3 at the 1000 ms mark. For the highest intensity of 103 dB, as noted before, there was very little (7%) decay over the duration of the experiment.

*Anopheles* males showed a nearly linear increase in flight speed response with sound intensity (Figs. 3c, 4b). In particular, the flight speed increased from about 5% over the baseline at 67 dB to 90% at 103 dB. For the lowest sound intensity of 67 dB, the maximum response was reached roughly 100 ms after the stimulus was initiated, but then decayed to baseline in about 350 ms.

*Anopheles* females (Figs. 3d, 4b) continued to show an extremely modest acoustotactic response at all sound intensities with a maximum, ~ 15% increase response in flight speed over baseline at 97 dB. Table 1 summarizes the key measures of the acoustotactic response in the current study.

**Table 1:** Summary of the acoustotactic response of *A. aegypti* and *An. gambiae* mosquitoes.

	Effective frequency range (Hz)	Most effective frequency (Hz)	Largest flight speed increase (%)	Time to maximum response (ms)	Effective intensity range (dB)
Male Aedes	100–800	250 ± 50	+ 130	~ 200	≳ 67
Female Aedes	100–300	200	+ 110	~ 200	≳ 79
Male Anopheles	100–500	400	+ 90	~ 100	≳ 79
Female Anopheles	200	200	+ 30	~ 100	≳ 97

For each test condition, the non-normalized (i.e. absolute) average flight speed against time is included in the online supplementary material (Figs. S1–S8). We also provide 4 videos (8.3 times slower, one video per mosquito group) to demonstrate the change in mosquito flight upon exposure to sound.

## Discussion

In this study, we successfully demonstrated and estimated, for the first time, the acoustic response of groups of single-sex, free-flying mosquitoes to incidental sound. We quantitatively showed that both male and female *Aedes* as well as *Anopheles* exhibited acoustotactic reactions to sound frequencies and intensity, and found the acoustic frequency ranges that cause the reaction, and the degrees of reaction are different in between mosquito species and genders.

Both male and female *Aedes* exhibited a robust response to the acoustic stimulus. For males, it was observed for frequencies between 100 and 800 Hz with the largest response at around 250 ± 50 Hz. For *Aedes* females, the response was observed over a narrower frequency range from 100 to 300 Hz, with the maximum occurring at 200 Hz. A detailed study of the sensitivity of the antennae and JO of *Aedes* to incident sound was carried out by Gopfert, et al.^27^ where they found that female and male *Aedes* were most sensitive to pure tones in the ranges 219–263 Hz and 344–406 Hz, respectively. Meanwhile, another study by Menda et al.^28^ demonstrated that *Aedes* is sensitive to sound frequencies of 150–350 Hz. Our observed response for females is therefore consistent with a JO-mediated trigger that senses and initiates locomotory reactions. For *Aedes* males however, while the range of response observed in our study includes that indicated by Gopfert, et al.^27^ we recorded a peak response at a frequency which is slightly lower than that previously reported. The reason for this discrepancy is not clear, but could be related to the sound intensity, which was much higher in our experiments compared to those employed by others.

It has been shown that mosquito auditory sensing is associated with precopulatory behavior where males and females use sensing of wing-tones to identify conspecifics and employ modulation of their own wing-tones to indicate sexual interest^2–7^. The typical WBFs of *Aedes* males and females are in the range of 650–700 Hz and 445–475 Hz^27^, respectively. For *Aedes* females, the frequency range of the increased flight speed-reaction resolved in the current study does not overlap with either the wing-tones of the females or the males, but the large frequency range of sensitivity observed for *Aedes* males does overlap with both these frequency ranges^7,19,29^. Therefore, at least for the Aedes females, it is not clear if the acoustotactic response observed here is connected with precopulatory behavior.

Although *Aedes* females showed a significant response to the imposed stimuli, we observed a greater acoustotactic response for *Aedes* males (higher normalized peak velocities, larger range of frequencies for response and lower threshold of sound intensity for acoustotactic response) as compared to females. From a physiological point of view, there are significant sex differences in the morphologies of the antennae, where the antennal flagellum of male mosquito is much more “plumose” and therefore likely more sensitive to acoustic perturbations than female mosquitoes^27,30^. Indeed, Gopfert et al.^27^ have shown that over a range of intensities of sound stimuli, the male antenna moves 1.4–1.5 times faster than the female antenna. Thus, our observations regarding the stronger acoustotactic response of males are consistent with the observations and experimental data of Gopfert et al.^27^. It has also been shown that acoustic sensing plays a more important role in the precopulatory behavior of male mosquitoes both in male–female and male-male interaction^31^ and therefore, the higher sensitivity of males to acoustic stimulus is in line with our understanding of mosquito precopulatory behavior.

*Anopheles* showed highly divergent responses in males versus females. While males exhibited a significant response at frequencies centered in a narrow band around 400 Hz, females seemed to be relatively unaffected. In fact, the highest intensity sounds seemingly elicited little to no effect. The typical WBFs of *An. gambiae* males and females are in the 650–700 Hz and 400–450 Hz ranges respectively^3,6^ and both male and females have been shown to respond to wing-tones of conspecifics of the other sex^3,6^. The peak response of *Anopheles* males in the current study is well-matched with the WBF of females, suggesting that the acoustotactic response of males is correlated with the acoustic sensing associated with precopulatory female-seeking behavior. The unresponsiveness of *Anopheles* females to intense sounds across the 100–1000 Hz frequency range was, however, unexpected, given that previous studies^3,6^ have clearly shown that *Anopheles* females are able to sense and respond to male wing-tones. Note that we believe the little response of female Anopheles is not due to deaf caused by high intensity sound. Female anopheles and female Aedes have similar antennal structures, if female anopheles were deaf due to high intensity sound, we would expect female Aedes were deaf (no response) as well, which is not the case. In addition, the test is conducted from low acoustic to high acoustic intensity, while we did not observe any response of female Anopheles for those low intensity conditions.

The response of the *Anopheles* females to acoustic stimulus was also different from that of *Aedes* females, suggesting significant species-specific variability. We noted measurable differences in the time-to-peak response for the two species with *Anopheles* exhibiting peak responses twice as fast (100 ms, male only) compared to *Aedes* (200 ms, male and female). This is likely associated with the latency of the afferent auditory neural system, the efferent neural system that sends signals to the flight muscles, the mechanical response time of the flight apparatus and/or finally, the time taken by changes in wing flapping to generate acceleration in the insect. The lattermost factor is expected to be the largest contributor in the time-to-peak response. However, this depends linearly on the mass of the insect as well as the changes in wing kinematics induced as a result of the sound stimulus^32,33^. Neither of these were measured in our experiments. Thus, at this point, this response time cannot be attributed to any particular factor with certainty. An accurate quantification of the neural response and flight muscle contractions, insect mass and flight forces, and wing size and kinematics would be essential to decode the physiology behind the observed response time in the acoustotactic response of these mosquitoes^34^. Flow field around the wing and antenna during acoustic stimulus and the corresponding mechanosensory mechanisms would also be essential to understand the physiology behind our findings and inspire future micro air vehicle designs^35^.

Finally, it is useful to compare the acoustotactic response observed in this study to the “startle” response observed in previous studies of tethered mosquitoes^2,5^. These experiments were conducted on *Toxorhynchites brevipalpis*, employed low intensity acoustic signals, and focused on the response of wing-tones of conspecifics on flapping behavior. The startle response is characterized by a transitory change of 30–60 Hz change in the WBF which decays to baseline in 1–2 s. Here, we observed that for the lowest sound intensities or for sound frequencies away from the peak response, the acoustotactic response of *Aedes* males and females and *Anopheles* males indeed decayed back to the baseline in less than 1 s. However, for highest intensity stimulus at the most sensitive frequencies, the decay over 1 s was much slower or even absent (for *Aedes* females). These results suggest that the response observed here could be an exaggerated version of the startle response, which for high sound intensities at sensitive frequencies can be prolonged beyond a few seconds, and possibly, even longer. It would be interesting to observe and record mosquito flight, precopulatory and mating behavior after the acoustic stimulus over a duration but this was not possible in the current setup due to the limitations of our camera memory.

## Methods

### Mosquito rearing

*Aedes aegypti* and *Anopheles gambiae* were reared and kept in the insectary at the Johns Hopkins Malaria Research Institute at a constant temperature of 77° ± 1° Fahrenheit and humidity of $84\pm 5$ %. The insects were exposed to light from 9:30 AM to 5:30 PM daily. Mosquitoes were manually separated based on sex and placed into separate 8″ × 8″ × 8″ cages shortly after pupation. They were fed a 10% sugar solution throughout the adult lifecycle in their cages. Tests were performed from 3 to 6 PM on days 5–8 after adult mosquitoes emerged, since earlier studies have shown that mosquitoes are quite active during dusk^36–39^.

### Experimental setup

**Figure 5:**
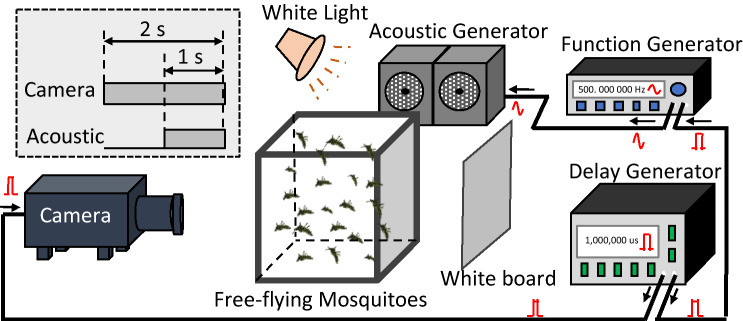
Test setup for the measurement of mosquito movement in response to acoustic waves. A high-speed camera recorded mosquito flight for 2 s, and sound triggered 1 s after the recording was initiated.

The setup is illustrated in Fig. 5. Sinusoidal acoustic waves were generated by combining a function generator (HP Hewlett-Packard 33120A) and a high-power speaker (Kanto YU2). There were typically about 25 flying mosquitoes in each cage, and the mosquitoes were back lighted by a white board, which reflected a 250 W halogen lamp diffusely. Mosquito flight was recorded by a high-speed CMOS camera (IDT Y4-S1, 1016 × 1016 pix, 13.68 μm/pixel) for 2 s at a frame rate of 500 FPS with an exposure time of 350–570 μs. Based on a previous study^4^, we estimated that given such mosquito flight speed, number density, camera resolution, frame rate higher than 500 fps would result in success of tracking. The high-speed camera and function generator were synchronized by a custom-made trigger system with temporal resolution down to 1 ns. A function generator, trigged by the synchronizer, sent a sinusoidal signal to the speaker, precisely 1 s after the camera recording was initiated. The amplitude of the sound was measured by a calibrated decibel meter (Tacklife MLM02). Tests were performed in a closed room with a background noise level of ~ 63 dB. It would be ideal if this test could be performed in a room with cotton gauze walls to eliminate any echo, like the study done by Menda, et al.^28^ when studying individual mosquito antennal neural response. For studying the response of a group of free-flying mosquitoes, the mesh cage will generate echoes and cannot be eliminated. When echo happened on the mesh of the wall, it is equivalent to the acoustic turned on from both side of the cage. Nevertheless, we believe the different responses between mosquito genders and species this study observed should not be affected.

### Test conditions

In order to characterize the sensitivity of the acoustotactic response to sound, the frequency was varied from 100 to 1000 Hz in100 Hz increments at a fixed decibel level of 103 dB (without weighting filter) measured 12.5 cm from a centrally-located speaker. The 12.5 cm is an experimental design choice driven by the size of the facility where the experiments were carried out. It might constrain the flight of mosquitoes and acoustic might bounce back from the mesh wall, while we believe the difference between genders and species this study observed should not be affected. We picked the highest acoustic intensity to study the effect of sound frequency to ensure a high likelihood of observing a measurable response. To quantify the magnitude of sound intensity on the flight response, we varied the sound intensity level from 67 to 103 dB at a fixed frequency of 200 Hz for females and 400 Hz for males. The air flow velocity propagated by the acoustic pressure is estimated around 0.1–7 mm/s^28^, since it is three orders smaller than the flight speed of the mosquitoes, the effect of wind gust due to acoustic is minimum.

One of the objectives of this study was to compare the response between *A. aegypti* and *An. gambiae*. To make the comparison meaningful, it was necessary to select the same frequency between the two species in the “effect of sound intensity” study. Since the female of *An. gambiae* only responded at 200 Hz while the female of *A. aegypti* responded between 100 and 300 Hz, we selected 200 Hz as common test condition for studying the effect of sound intensity on female mosquito startle response. Similarly, 400 Hz was selected for studying the effect of sound intensity on male mosquito response. In each test condition, 6 independent experiments were performed, and each group of mosquitoes was given enough time (> 10 min) to recover between recordings. In each experiment, the camera filmed at 500 FPS for 2 s. Each test was performed for each species. Note the overlapped conditions between Tables 1 and 2 (103 dB, 200 Hz for female and 400 Hz for male) were tested only one time.

**Table 2:** Test conditions for the study of acoustic wave frequency effect on mosquito flight.

	Decibel (dB)	Acoustic wave frequency (Hz)									
Female	103	100	200	300	400	500	600	700	800	900	1000
Male	103	100	200	300	400	500	600	700	800	900	1000

### Data processing

Each test condition listed in Tables 2 and 3 yields 6 video files since the experiment was repeated 6 times. These high-speed videos of free-flying mosquitoes were post processed by an in-house MATLAB script. First, each frame of the video was extracted and subtracted from background; second, individual mosquitoes were recognized based on the “regionprops” function available in MATLAB; third, by repeating step 1 and 2, we obtained the trajectory of the mosquitoes; lastly, each mosquito was tracked using a 4-frame particle tracking algorithm^40^, and the in plane velocity (${V}_{x}, {V}_{y}$) of each mosquito was determined.

**Table 3:** Test conditions for the study of acoustic wave Decibel effect on mosquito flight.

	Frequency (Hz)	Acoustic wave decibel (dB)					
Female	200	67	79	85	91	97	103
Male	400	67	79	85	91	97	103

**Figure 6:**
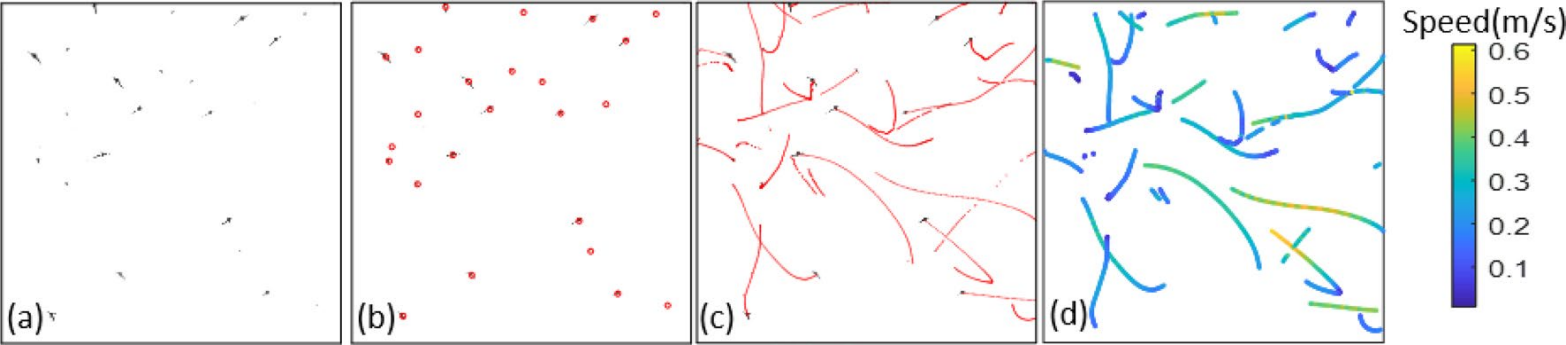
Demonstration of videogrammetry based estimation of flight speeds of free-flying mosquitoes. (**a**) and (**b**) are individual snapshots of a mosquito group and mosquito location identification results, respectively. (**c**) and (**d**) are mosquito trajectories composed of male *Aedes* over 100 frames without and with flight speed information, respectively.

Figure 6 shows the above procedure for a group of Aedes males. Figure 6a shows one snapshot of the free-flying mosquito group after background subtraction. Figure 6b shows the result of identification of mosquito locations. Figure 6c shows mosquito trajectories over 100 frames. Figure 6d shows mosquito speeds along with trajectory over the 100 frames.

Following the procedure demonstrated in Fig. 6, for each frame, the average flight speed of the mosquitoes in the group was estimated as Flight speed $= \sqrt {\left( {V_{x}^{2} + V_{y}^{2} } \right)}$. This flight speed information was obtained over 2 s at the time interval of 2-ms. We then ensembled averaged results from the six independent tests and presented means ± standard deviations. Note that since the imaging is two-dimensional, the estimated flight speed underestimated the true speed since the velocity vector in the third dimension was not captured. Assuming that the mosquitoes have an equal probability of flying in any direction, it is expected that the true average speed is larger by about a factor of $\sqrt{3/2}$=1.22 than that estimated in the current experiments. However, we based our analysis on the *relative change* in the velocity before and after the acoustic stimulus is introduced, and this normalized velocity is expected to be unaffected by the underestimation of the true speed.

## Supplementary Information

Supplementary Information.
