# Supplementary material for: Acoustotactic response of mosquitoes in untethered flight to incidental sound
Source: Sci Rep. 2021 Jan 21;11:1884. doi: 10.1038/s41598-021-81456-5 (PMC7820424; doi:10.1038/s41598-021-81456-5)
Supplement: Supplementary file 1 — Supplementary Information. [file 41598_2021_81456_MOESM1_ESM.zip › supplementary material/SupplementaryMaterial_RawDataPlots.pdf]

Supplementary mater of "Acoustotactic Response of Mosquitoes in Untethered Flight to Incidental Sound"

Zhongwang Dou[1], Aditi Madan[2], Jenny S. Carlson[3], Joseph Chung[1], Tyler Spoleti[1], George Dimopoulos[3], Anthony Cammarato[2], Rajat Mittal[1]

1. Department of Mechanical Engineering, Whiting School of Engineering, Johns Hopkins University Baltimore, MD, USA

2. Division of Cardiology, Department of Medicine, School of Medicine, Johns Hopkins University, Baltimore, MD, USA

3. Department of Molecular Microbiology and Immunology, Bloomberg School of Public Health, Johns Hopkins University, Baltimore, MD, USA

Correspondence to R.M. (email: mittal@jhu.edu)

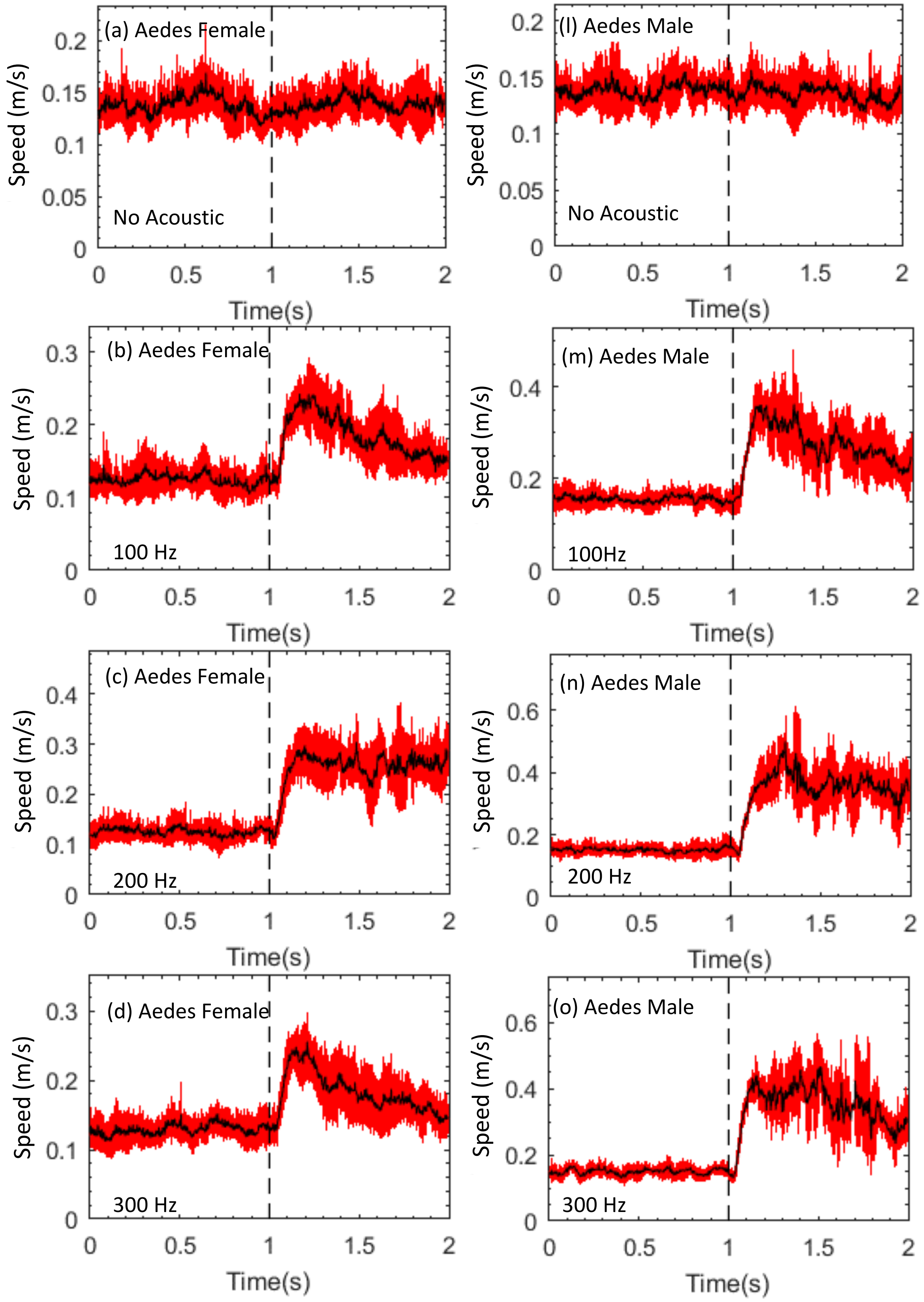

(a) Aedes Female
No Acoustic
(l) Aedes Male
No Acoustic
(b) Aedes Female
100 Hz
(m) Aedes Male
100Hz
(c) Aedes Female
200 Hz
(n) Aedes Male
200 Hz
(d) Aedes Female
300 Hz
(o) Aedes Male
300 Hz
Speed (m/s)
Time(s)

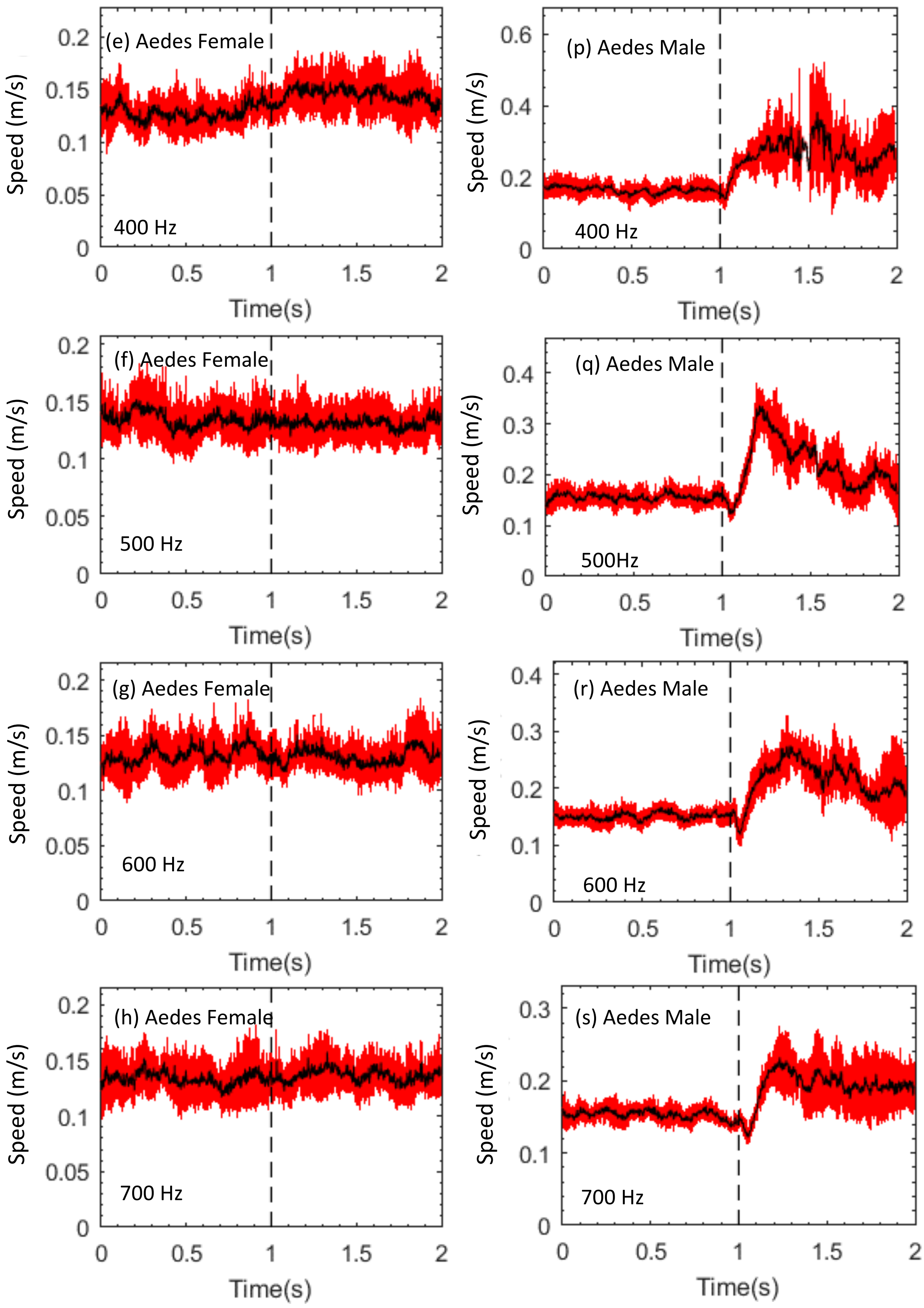
(e) Aedes Female
400 Hz
(p) Aedes Male
400 Hz
(f) Aedes Female
500 Hz
(q) Aedes Male
500Hz
(g) Aedes Female
600 Hz
(r) Aedes Male
600 Hz
(h) Aedes Female
700 Hz
(s) Aedes Male
700 Hz
Speed (m/s)
Time(s)

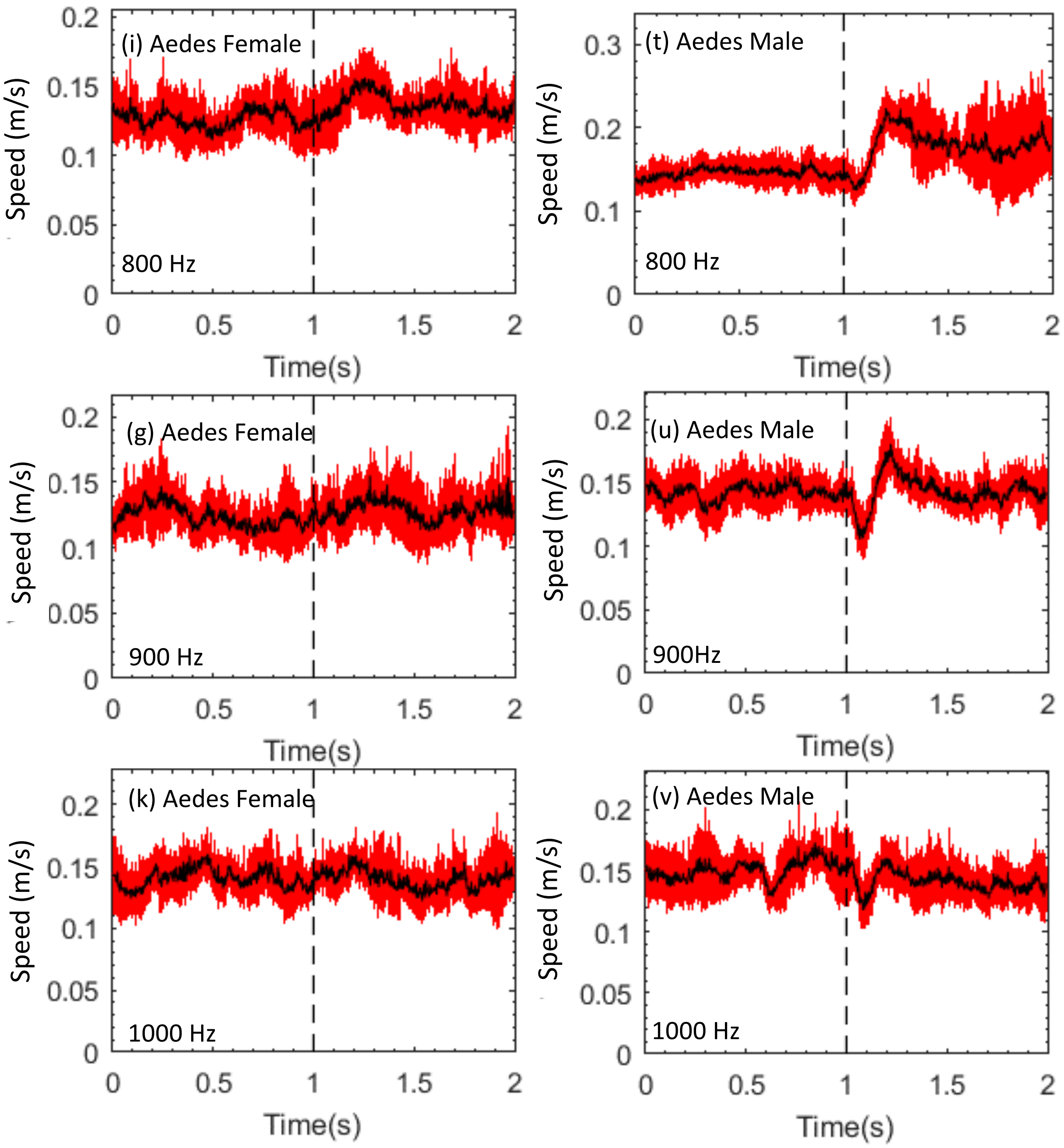

FIGURE S1. Raw data for Aedes when frequency was swept.

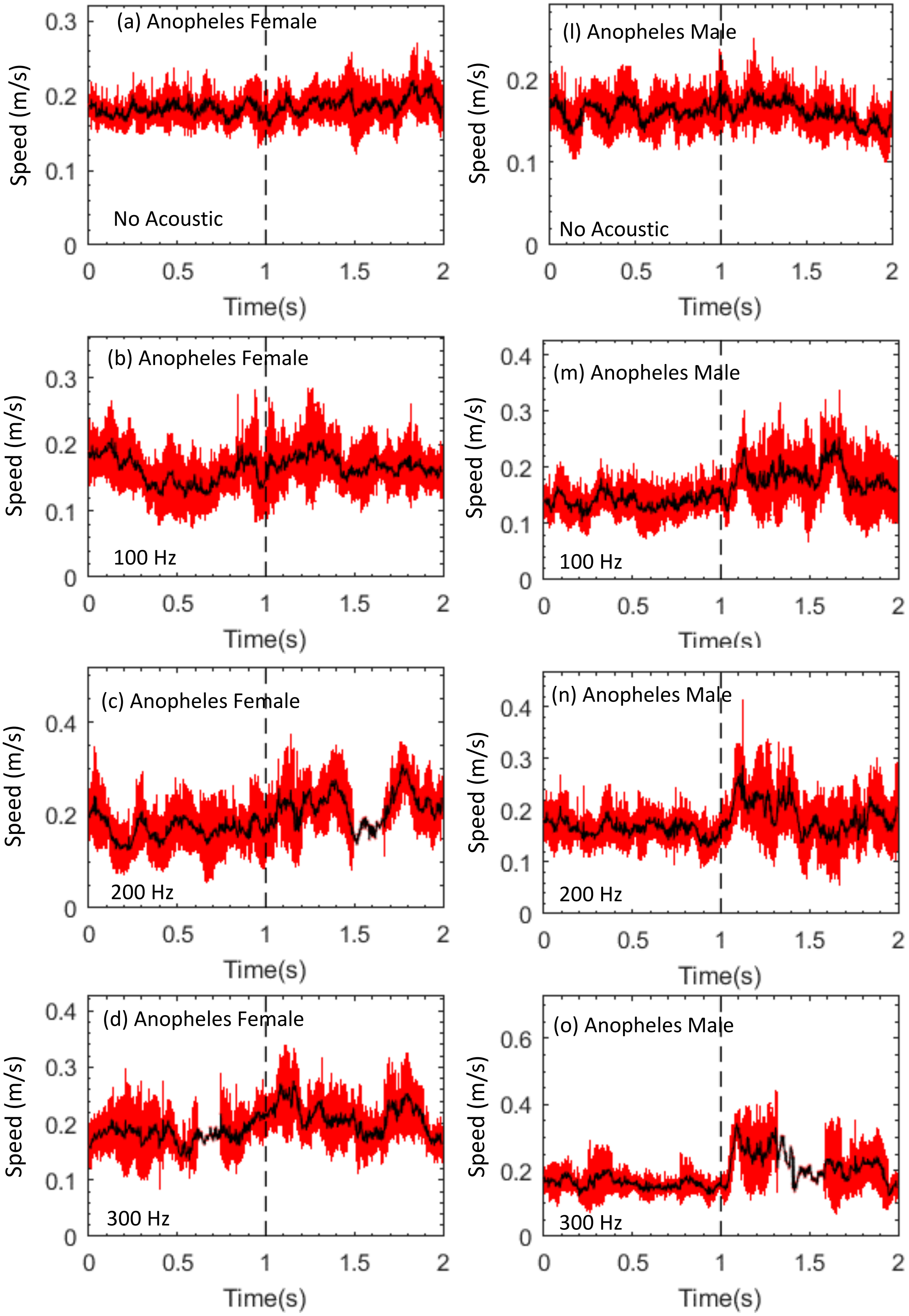

(a) Anopheles Female
No Acoustic
(l) Anopheles Male
No Acoustic
(b) Anopheles Female
100 Hz
(m) Anopheles Male
100 Hz
(c) Anopheles Female
200 Hz
(n) Anopheles Male
200 Hz
(d) Anopheles Female
300 Hz
(o) Anopheles Male
300 Hz
Speed (m/s)
Time(s)

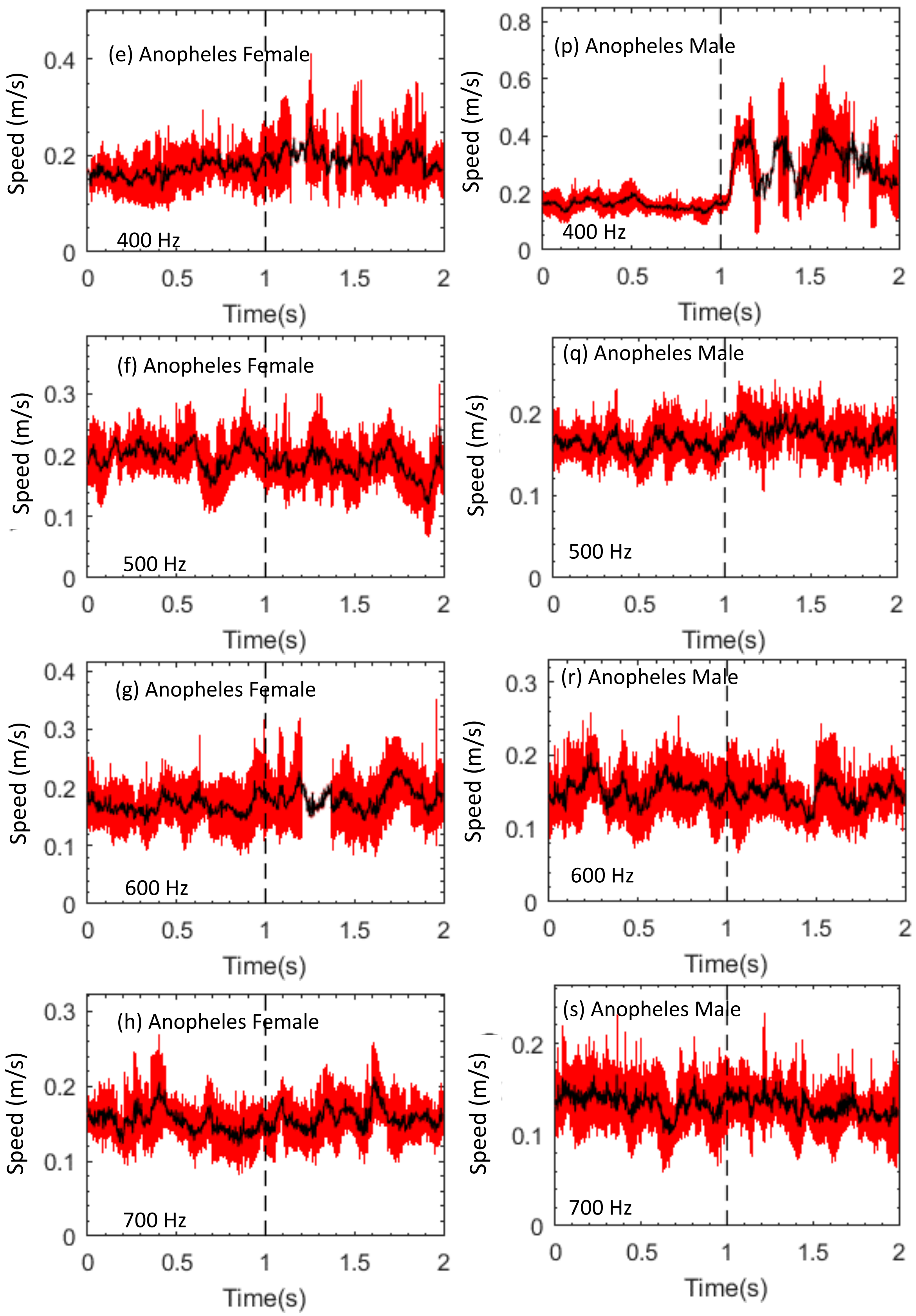

(e) Anopheles Female
400 Hz
Speed (m/s)
Time(s)
(p) Anopheles Male
400 Hz
(f) Anopheles Female
500 Hz
(q) Anopheles Male
500 Hz
(g) Anopheles Female
600 Hz
(r) Anopheles Male
600 Hz
(h) Anopheles Female
700 Hz
(s) Anopheles Male
700 Hz

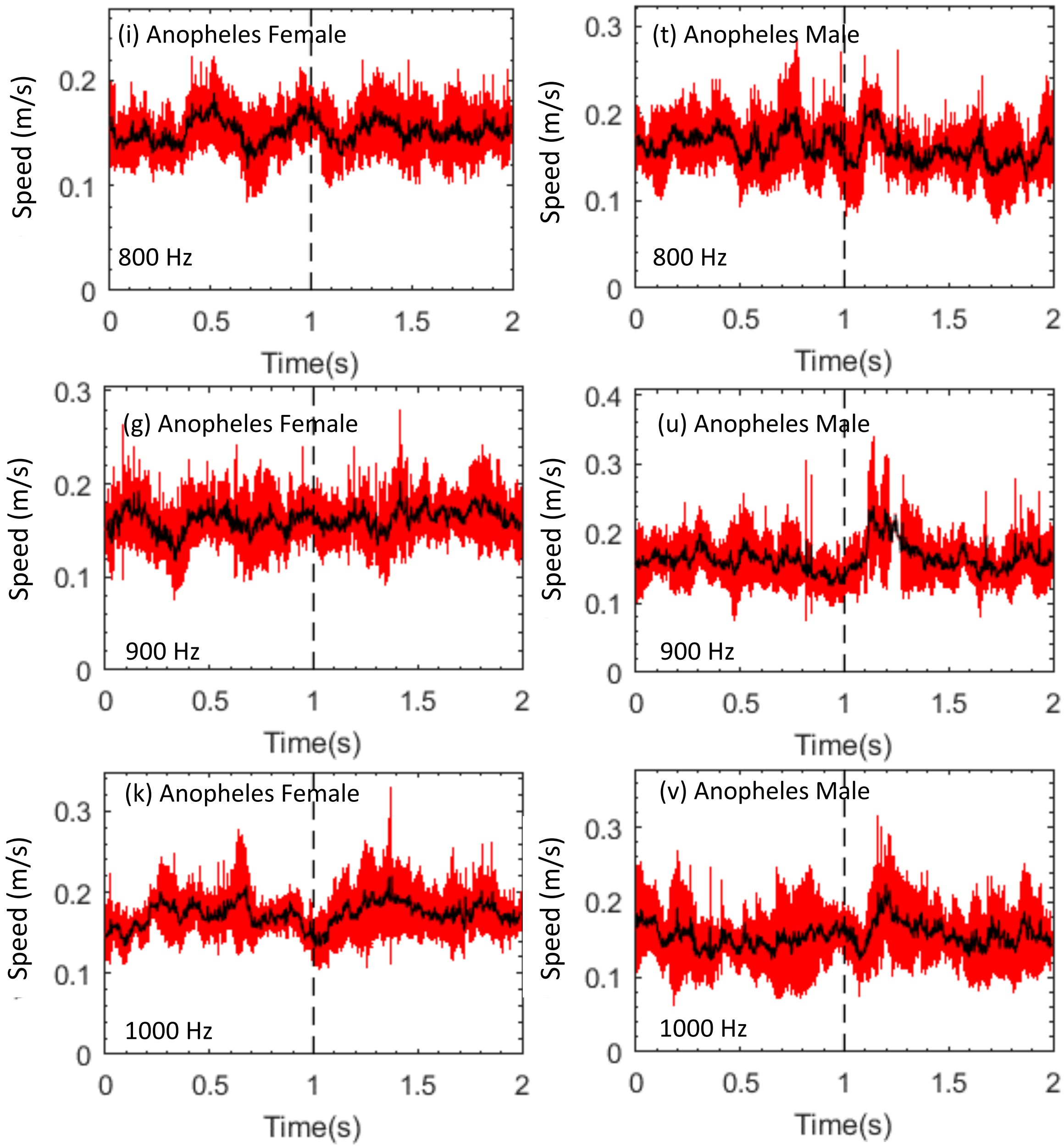

FIGURE S2. Raw data for Anopheles when frequency was swept.

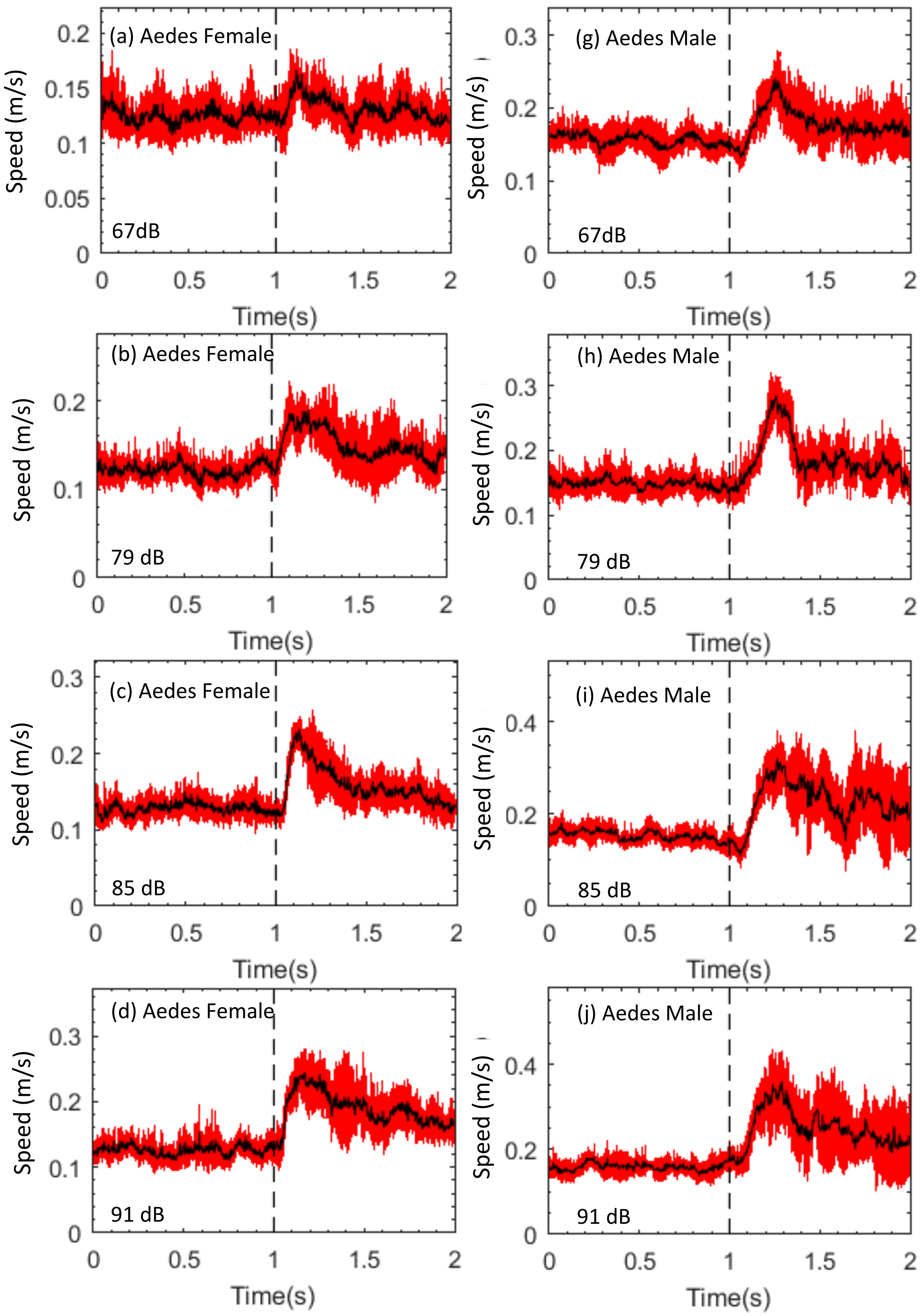

(a) Aedes Female
67dB
(b) Aedes Female
79 dB
(c) Aedes Female
85 dB
(d) Aedes Female
91 dB
(g) Aedes Male
67dB
(h) Aedes Male
79 dB
(i) Aedes Male
85 dB
(j) Aedes Male
91 dB
Speed (m/s)
Time(s)

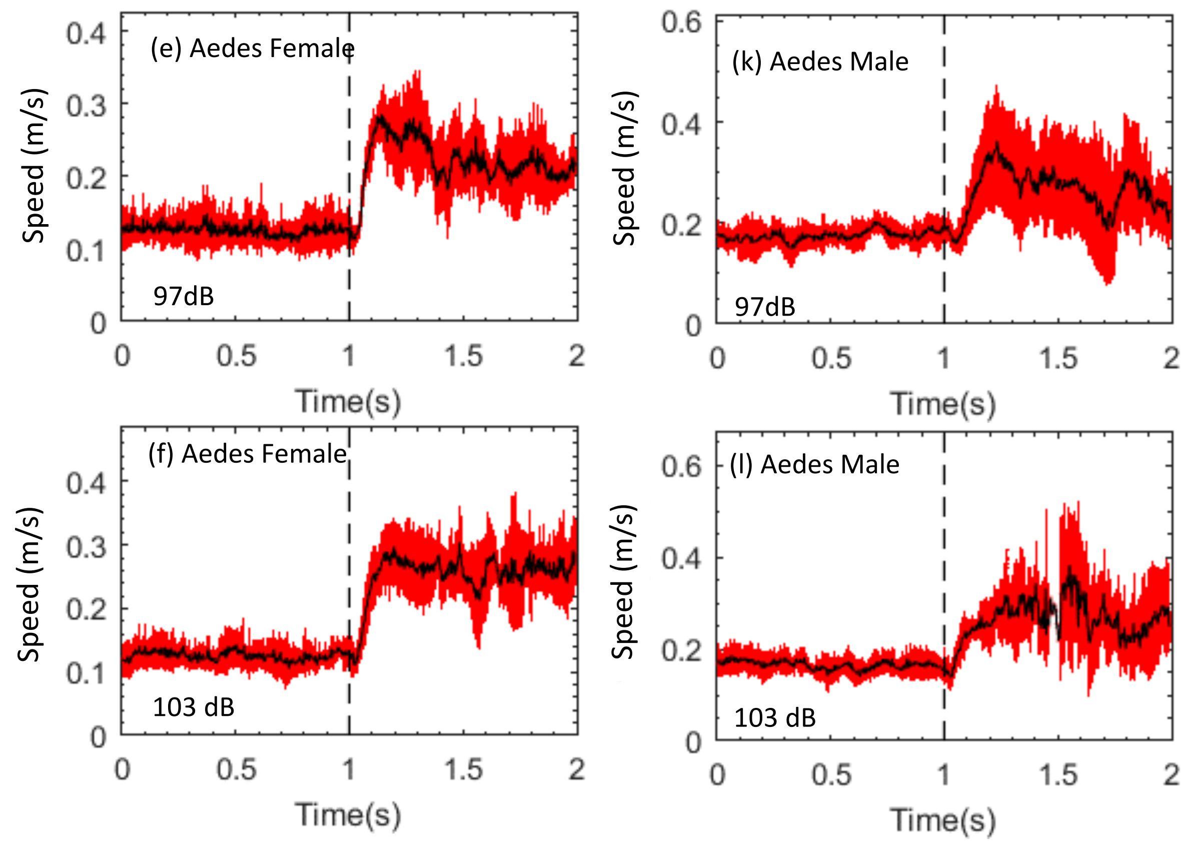

FIGURE S3. Raw data for Aedes when acoustic intensity was swept.

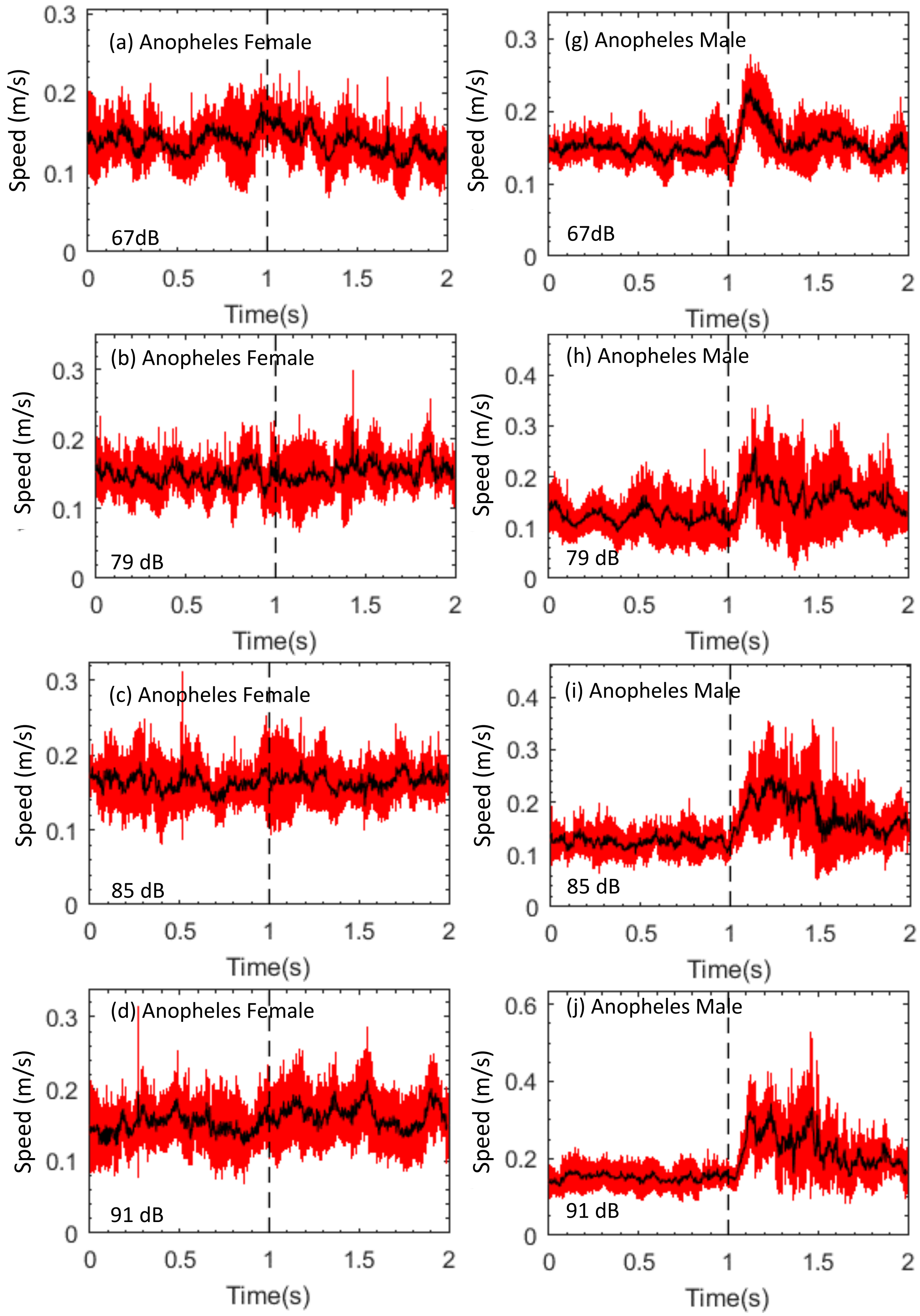
(a) Anopheles Female
67dB
(b) Anopheles Female
79 dB
(c) Anopheles Female
85 dB
(d) Anopheles Female
91 dB
(g) Anopheles Male
67dB
(h) Anopheles Male
79 dB
(i) Anopheles Male
85 dB
(j) Anopheles Male
91 dB
Speed (m/s)
Time(s)

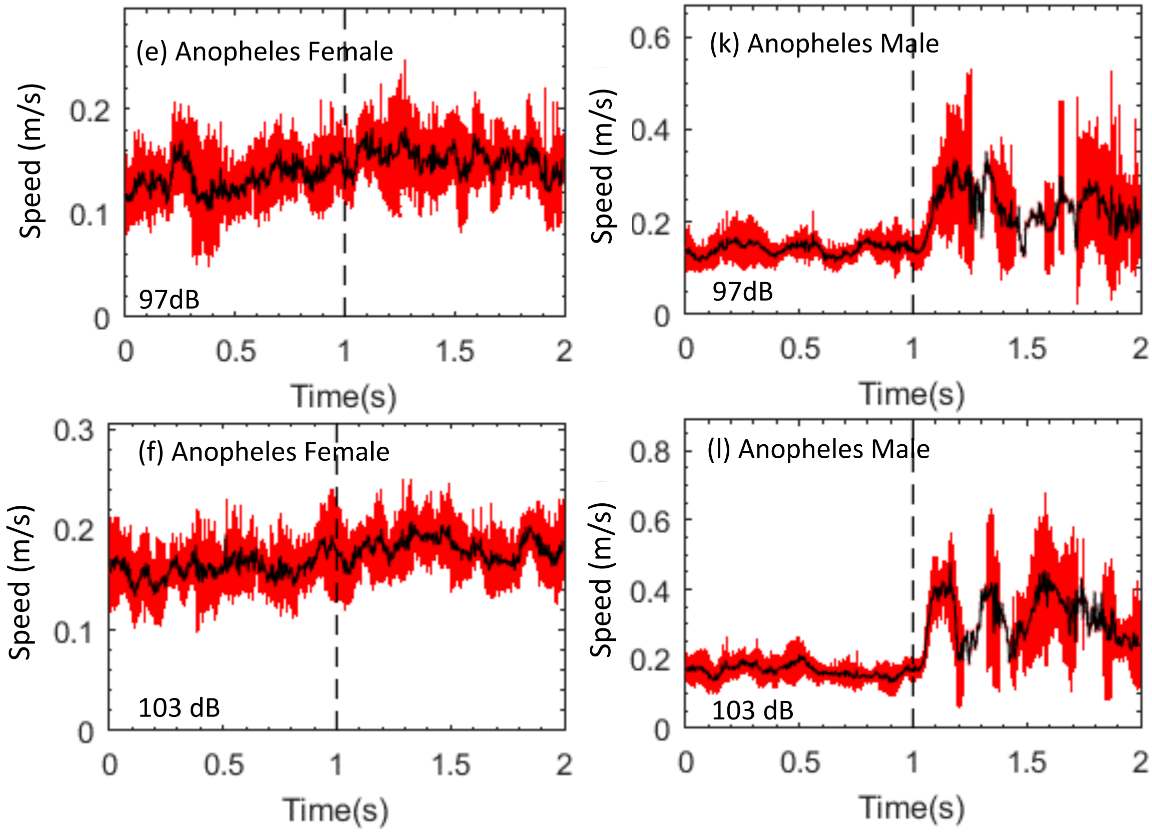

FIGURE S4. Raw data for Anopheles when acoustic intensity was swept.
